# An outcome of educational intervention on the menstrual hygiene practices among school girls in Ogun State, Nigeria: a quasi-experimental study

**DOI:** 10.11604/pamj.2021.40.214.30601

**Published:** 2021-12-08

**Authors:** Catherine Olukemi Agbede, Ugochinyere Chinasa Ekeanyanwu

**Affiliations:** 1 Department of Public Health, Babcock Unversity, Ilishan Remo, Ogun State, Nigeria

**Keywords:** Adolescent girls, menstrual hygiene, practices, peer-led, parent-led, health belief model

## Abstract

**Introduction:**

adolescence is a special period of physical and psychological preparation for safe motherhood that requires specific attention as it marks the onset of menarche. Hygiene-related practices are significant during menstruation to prevent being predisposed to reproductive tract infections and other complications. The main objective of the study was to determine the outcome of educational intervention on the menstrual hygiene practices among school girls in Ogun State, Nigeria using the health belief model. One hypothesis guided the study.

**Methods:**

the study utilized a quasi-experimental design comprising of three experimental groups and one control group. The population of the study was one hundred and twenty (120) in-school adolescent girls. The multistage sampling technique was used in selecting participants from four secondary schools within Ogun State. A validated semi-structured questionnaire was used for data collection with a Cronbach alpha with a score of 0.87. Data collected were analysed using Statistical Package for Social Sciences (SPSS) version 23.

**Results:**

a paired T-test analysis was conducted in determining the results. The observed difference in the mean in the parent-led (Δ = 3.80; t = 20.886; p=0.000), peer-led group (Δ = 4.23; t = 19.901; p=0.000), and combination of parent and peer-led group intervention group (Δ = 3.53; t = 18.015; p=0.000) were statistically significant.

**Conclusion:**

peer-led experimental group had the greatest effective change in the level of adolescent girls´ menstrual hygiene practices. Peer educators could be recruited for future interventions and scaled up in other aspects of young girls´ reproductive health and well-being.

## Introduction

Adolescence is a special period of physical and psychological preparation for safe motherhood that requires specific attention as it marks the onset of menarche [1]. According to the United Nations Children Fund (UNICEF), an adolescent is defined as any person between the ages of 10-19 years [2]. Globally, about 1.2 billion adolescents are of this age and 85% live in developing nations [2,3]. During menstruation, hygiene-related practices are fundamental particularly at the onset [4]. Good hygienic practices require the use of sanitary pads, washing of the body and genital areas, frequently changing of menstrual absorbents as well as proper disposal of used menstrual absorbents [5]. This will enhance the confidence of females in various aspects [6]. On the other hand, if proper hygiene is not practiced during a menstrual period, it could lead to poor academic performance, school dropout, and predispose one to reproductive tract infections (RTIs), pelvic inflammatory diseases, and other complications like carcinoma of the cervix and infertility problems [5,6]. Currently, the global prevalence of RTIs and their complications is unacceptably high between 15-80% in most impoverished communities [7]. Research has shown that women and adolescents girls with poor menstrual hygiene are three times more likely to have RTI than those with proper and adequate menstrual hygiene [8]. The World Health Organisation (WHO) acknowledges that about 80% of the global burden lies in low- and middle-income countries especially in the rural settlements as a result of ignorance and inadequate support for managing menstruation [9,10].

In Nigeria, over 10 million children mostly adolescent girls are already out of school due to access to adequate safe menstrual hygienic products and approximately 25 percent lack adequate privacy for defecation and menstrual hygiene management [11,12]. Researchers have discovered between 31 and 56% of schoolgirls in Nigeria using toilet tissue, cotton wool, or cloth to absorb menstrual blood as opposed to sanitary pads [13]. Learning about menstrual hygiene-related practices is a significant part of health education for adolescent girls [14]. This could enable them to continue to work and keep up with hygienic habits during their adult life. It will also help to improve maternal health; which can have an impact on the sustainable development goals (SDGs) for example SDG 3 [14]. The Health belief model (HBM) was espoused for this study. This model states that a stimulus or cue to action must be present to trigger the health-promoting behaviour [15]. However, this has been used in assessing underlying menstrual hygiene-related practices of adolescent girls and also has been used to inform the development of interventions to improve health behaviours [16,17]. Hence, this study needed to be conducted to determine the outcome of educational intervention on the menstrual hygiene practices among school girls in Ogun State, Nigeria. The study postulated that there will be no significant difference in the level of menstrual hygiene practices among in-school female adolescents between the baseline and 6-weeks follow-up.

## Methods

**Study design:** the study was a school-based, quasi-experimental study comprising of three experimental groups and a control group using a quantitative approach. The three experimental groups were peer-led, parent-led, and a combination of both. The study encompassed a health education programme that was based on menstrual hygiene-related practices. The health education programme was delivered to the experimental groups and a placebo on COVID-19 prevention was delivered to the control group. Data were collected across the four groups before the program and at 4 weeks´ immediate post-intervention as well as at 6-weeks follow-up.

**Study area and population:** the present research was conducted in a rural area of Ogun State. Ogun State is located in the South-West and is situated between latitude 6.2°N and 7.8°N, and longitude 3.0°E and 5.0°E. It has an estimated 2016 population of 7.2 million and is predominantly covered by tropical rainforest and has a wooded savannah in the Northwest. There are about two hundred and ninety-one (291) public secondary schools and one hundred and eleven owned private secondary schools. The tertiary institutions both public and private are about twenty-one (21) making it the highest in the country. There are also twenty local government areas in Ogun State consisting of cities, towns, and villages. The study population comprised of in-school adolescent girls aged 10-19.

**Inclusion and exclusion criteria:** in-school adolescent girls in a rural area who attained menarche and were between the ages of 10-19; who were present on the day of the study and whose parents gave consent and were willing to take part in the study. In-school adolescent girls who are not in a rural area who did not attain menarche; who were below and above 10-19 years of age and whose parents did not give consent and were not willing to take part in the study.

**Sampling technique:** the multistage sampling technique was used in selecting the participants. A senatorial district in Ogun State was purposively selected; from the senatorial district, four local government areas were randomly selected after which a public secondary each was randomly chosen through balloting from the selected local government areas.

**Sample size:** a total of one hundred and twenty adolescent girls were selected for the study. The sample size was derived from the computation using a level of significance of 95% and 80% power. There was no given estimate of the prevalence of poor menstrual hygiene in secondary school female adolescents in Ogun State. Thus, the sample size was determined using the prevalence at 50%. The sample size was derived from the computation using a level of significance of 95% and 80% power. There was no given estimate of the prevalence of poor menstrual hygiene in secondary school adolescent girls´ in Ogun State.

Thus, the sample size was determined using the prevalence at 50%.


$$n=\frac{{\left(Z_{\alpha }+Z_{\beta }\right)}^{2}\times P_{0}(1-P_{0})}{{\left(P_{1}-P_{0}\right)}^{2}}$$

Where n is the minimum sample size per group Zα = 95% confidence interval i.e. 1.96; Zβ = 80% i.e. 0.84 (power to detect changes in the outcome variable and avoid type II error) P0 = prevalence (at 50%); P1 = 80% (desired level of outcome variable);


$$N=\frac{{\left(1.96+0.84\right)}^{2}\times 0.5(1-0.5)}{{\left(0.8-0.5\right)}^{2}}=\frac{{\left(2.8\right)}^{2}\times 0.5(0.5)}{{\left(0.8-0.5\right)}^{2}}=\frac{7.84\times 0.25}{0.09}=21.78≃22$$

The minimum sample size was 22. Ten percent was added to the minimum sample size to take care of attrition. The total number of participants after adding 10% of 22 was 22 + 2.2 = 24≃30. Based on the computation a total number of 120 participants (30 per group x 4) was considered for the study (representing 30 people per group). Instrument for data collection. A semi-structured questionnaire made up of multiple-choice questions that sought information regarding socio-demographic characteristics and menstrual hygiene practices of participants was utilized for the study. The semi-structured questionnaire was designed after a review of the literature [18-21].

**Validity and reliability:** face validity of the instrument was adopted, and this was checked by faculty members. Content validity was also adopted by incorporating items identified in the literature review. To ascertain the reliability of the instrument, a pilot test was conducted for internal consistency of the instrument using 10% of the anticipated sample size using adolescent girls with the same characteristics. The data were statistically analyzed using the Cronbach alpha standard score to test its reliability. The reliability score generated was 0.87.

**Data analysis:** the data from the study were collated, entered, and coded using. Statistical Package for Social Sciences (SPSS) version 23. Data were presented using descriptive and inferential statistics and a statistical level of significance was set at p<0.05

**Ethical approval and informed consent:** ethical approval was obtained from Babcock University Health Research and Ethics Committee; the Ministry of Education Planning Research and Statistics, and the Ministry of Health Research Ethics, Ogun State. Written informed consent was obtained from the participants and their parents (mothers) explaining the aim of the research.

## Results

One hundred and twenty in-school adolescent girls were recruited from four junior and senior secondary schools in four local government areas in Ogun State, Nigeria. At baseline, the experimental groups and the control group each had an equal amount of 30 participants. The response rate across the four groups was 100% after the health education intervention was administered. There were more in-school adolescent girls in the middle adolescence (14-16 years) age bracket across the three experimental groups. The overall mean ± SD age of the adolescent girls recruited into this study was 14.92 ± 1.82 years and the mean ± SD age at menarche in this study was 12.73 ± 1.38 years. The adolescent girls were recruited from all secondary classes except SS3. The majority were Christians and only a few were reported to be a traditionalist. The ethnic distribution showed that the majority of the adolescent girls were from the Yoruba ethnic group and a few from the Igbo, Hausa, and other ethnic groups. The findings also disclosed that the majority of adolescent girls´ mothers had at least secondary or tertiary education and were businesswomen or traders (Table 1). The majority of participants across the three experimental groups and the control group reported to have been aware of menstrual hygiene and the information was sourced from mothers, teachers, sisters, and relatives (Table 2).

**Table 1:** baseline distribution of socio-demographic characteristics of adolescents in control and intervention groups: age, age at menarche, class, religion, ethnic group, mother’s level of education and mother’s occupation

Variables	Parent-led N (%)	Peer-led N (%)	Parent-led and Peer-led N (%)	Control N (%)	p-value
**Age in years**					
10-13	9 (30.0)	7 (23.3)	12 (40.0)	5 (16.7)	0.880^b^
14-16	15 (50.0)	20 (66.7)	17 (56.7)	9 (30.0)	
17-19	6 (20.0)	3 (10.0)	1 (3.3)	16 (53.3)	
Total	30 (100)	30 (100)	30 (100)	30 (100)	
Mean±SD	14.83±1.91	14.77±1.48	13.93±1.48	16.17±1.70	
**Age at menarche**					
Mean±SD	12.97±1.47	13.07±1.70	12.07±1.17	12.83±0.87	0.190
Class					
JS 1	7 (23.3)	10 (33.3)	7 (23.3)	6 (20.0)	
JS 2	7 (23.3)	6 (20.0)	6 (20.0)	10 (33.3)	
JS 3	5 (16.7)	5 (16.7)	6 (20.0)	6 (20.0)	0.978^a^
SS 1	6 (20.0)	4 (13.3)	6 (20.0)	3 (10.0)	
SS 2	5 (16.7)	5 (16.7)	5 (16.7)	5 (16.7)	
Total	30 (100)	30 (100)	30 (100)	30 (100)	
**Religion**					
Christianity	23 (76.7)	22 (73.3)	20 (66.7)	18 (60.0)	
Islam	6 (20.0)	8 (26.7)	10 (33.3)	12 (40.0)	0.431^a^
Traditional	1 (3.3)	0 (0.0)	0 (0.0)	0 (0.0)	
Total	30 (100)	30 (100)	30 (100)	30 (100)	
**Ethnic group**					
Yoruba	26 (86.7)	26 (86.7)	24 (80.0)	27 (90.0)	
Igbo	0 (0.0)	4 (13.3)	6 (20.0)	1 (3.3)	0.330^a^
Hausa	1 (3.3)	0 (0.0)	0 (0.0)	2 (6.7)	
Others	1 (3.3)	0 (0.0)	0 (0.0)	0(0.0)	
Total	30 (100)	30 (100)	30 (100)	30 (100)	
**Mother's level of education**					
No formal education	8 (26.7)	3 (10.0)	5 (16.7)	6 (20.0)	
Primary	1 (3.3)	4 (13.3)	0 (0.0)	3 (10.0)	
Secondary	15 (50)	14 (46.7)	20 (66.7)	17 (56.7)	
Tertiary	6 (19.9)	9 (30.0)	5 (16.7)	4 (13.3)	0.553^a^
Total	30 (100)	30 (100)	30 (100)	30 (100)	
**Mother's occupation**					
Business/trading	19 (63.3)	24 (80.0)	26 (86.7)	25 (83.3)	
Housewife	4 (13.3)	2 (6.7)	1 (3.3)	2 (6.7)	
Farmer	4 (13.3)	1 (3.3)	2 (6.7)	1 (3.3)	0.321^a^
Teacher	2 (6.7)	1 (3.3)	1 (3.3)	1 (3.3)	
Others	1 (3.3)	2 (6.7)	0 (0.0)	1 (3.3)	
Total	30 (100)	30 (100)	30 (100)	30 (100)	

a p-value obtained by Chi-square test ;^b^p-value obtained by T-

**Table 2:** baseline distribution of female adolescents' menstrual hygiene awareness in experimental and control groups

Variables	Parent-led N (%)	Peer-led N (%)	Parent-led and peer-led N (%)	Control N (%)	p-value
**Heard about menstrual hygiene?**					
Yes	20 (66.7)	21 (70.0)	24 (80.0)	26 (86.7)	0.247a
No	10 (33.3)	9 (30.0)	6 (20.0)	4 (13.3)	
Total	30 (100)	30 (100)	30 (100)	30 (100)	
**Source of menstrual hygiene information, Not informed**					
Mother	10 (33.3)	9 (30.0)	6 (20.0)	4 (13.3)	0.009b*
Sister	12 (40.0)	6 (20.0)	13 (43.3)	18 (80.0)	
Friends	3 (10.0)	2 (6.7)	3 (10.0)	8 (26.7)	
Relative	0 (0.0)	3 (10.0)	1 (3.3)	0 (0.0)	
Teacher	0 (0.0)	2 (6.7)	2 (6.7)	0 (0.0)	
Media	4 (13.3) 1 (3.3)	8 (26.7) 0 (0.0)	5 (16.7) 0 (0.0)	0 (0.0) 0 (0.0)	
Total	30 (100)	30 (100)	30 (100)	30 (100)	

p-value obtained by Chi-square test *significant at p<0.05

**Baseline distribution of adolescents girls´ menstrual hygiene practices:** the menstrual hygiene practices of adolescent girls were measured on a 7-point rating scale. The responses were grouped and the results showed that across the four groups, the majority of the adolescent girls in the parent-led group, peer-led group, parent and peer-led group, and control group had a low level of practice of menstrual hygiene behaviors. The mean ± SD scores for each group at baseline were 2.10 ± 0.995, 1.70 ± 1.12, 2.47 ± 0.94, and 2.10 ± 1.30 (Table 3).

**Table 3:** comparison of adolescent girls practice of menstrual hygiene at baseline, post-intervention and 6^th^-week follow-up for experimental and control groups

Menstrual hygiene practice	Parent-led group N (%)	Peer-led group N (%)	Peer and parent-led group N (%)	Control N (%)	P-value
**Baseline**		Measured on a 7 point rating scale			
Low (0-3.5)	28 (93.3)	29(96.7)	27 (90.0)	26 (86.7)	
High (3.51- 7)	2 (6.7)	1(3.3)	3 (10)	4(13.3)	
Total	30 (100)	30 (100)	30(100)	30 (100)	0.067a
Mean±SD	2.10±0.995	1.70±1.12	2.47±0.94	2.10±1.30	
**Post intervention**		Measured on a 7 point rating scale			
Low (0- 3.5)	4 (13.3)	8 (26.7)	2(6.7)	26(86.7)	
High (3.51- 7)	26 (86.7)	22 (73.3)	28 (93.3)	4 (13.3)	
Total	30 (100)	30 (100)	30(100)	30 (100)	0.000
Mean±SD	5.32±0.62	5.70±0.54	5.87±0.73	2.23±1.22	
**6-weeks follow Up**		Measured on a 7 point rating scale			
Low (0-3.5)	0 (0.0)	0 (0.0)	0 (0.0)	25(83.3)	
High (3.51- 7)	30(100.0)	30 (100.0)	30 (100.0)	5(16.7)	
Total	30 (100)	30 (100)	30(100)	30 (100)	0.000
Mean±SD	5.90±0.55	5.93±0.25	6.00±0.37	2.30±1.21	

p-value obtained by oneway ANOVA' *significant at p<0.05

**The outcome of educational intervention of adolescent girls practice of menstrual hygiene:** the outcome of the intervention of in-school adolescent girls practice of menstrual hygiene was assessed at immediate post-intervention and the results showed changes in the mean scores across the four groups parent-led, peer-led, parent and peer-led intervention, and control groups were statistically significant at p= 0.000. The outcome was also assessed at 6^th^-week follow-up and the results showed that there were changes in the mean score across the four groups. The evaluation indicated that the adolescent girls in the three experimental groups scored between 3.51- and 7-points and the combination of parent and peer-led intervention groups recorded the highest mean score (Table 3).

**Change in the level of menstrual hygiene practices at baseline to 6-weeks follow-up:** at the end of the intervention, the peer-led group had a statistically significant change in the mean of the adolescent girls' level of menstrual hygiene practices. The observed mean difference was 4.23 (p = 0.000). In the parent-led group, the mean difference was 3.80 (p= 0.000); while the combination of both was 3.53 (p= 0.000). There was a decrease of 0.20 in the control group which was also statistically significant (Table 4).

**Table 4:** change in the level of menstrual hygiene practices at baseline to 6-weeks follow up

Groups	x̄ (SE)	SD	Mean difference	ES (CI)	df	t	p-value
**Parent-led intervention group**							
Baseline	2.10(0.18)	1.00	3.80	4.80 (4.60 -5.00)	29	20.886	0.000
6^th^-week follow-up	5.90(0.10)	0.55					
**Peer-led intervention group**							
Baseline	1.70(0.20)	1.12	4.23	5.30 (5.10 - 5.50)	29	19.901	0.000
6th-week follow-up	5.93(0.05)	0.25					
**Parent and peer-led intervention group**							
Baseline	2.47(0.17)	0.9	3.53	5.03 (4.85-5.20)	29	18.015	0.000
6^th^-week follow-up	6.00(0.07)	0.37					
control group							
Baseline	2.10(0.24)	1.30	0.20	0.62 (0.15-0.47)	29	0.711	0.483
6^th^-week follow-up	2.30(0.22)	1.21					

**Research hypothesis:** there will be no significant difference in the level of menstrual hygiene practices among in-school adolescent girls between the baseline and 6-weeks follow-up. To determine the results, a paired T-test analysis was conducted. The results showed that the observed difference in the mean in the parent-led (Δ= 3.80; t = 20.886; p= 0.000), peer-led group (Δ = 4.23; t = 19.901; p = 0.000), and combination of parent and peer-led group intervention group (Δ = 3.53; t = 18.015; p= 0.000) were statistically significant (Table 4). The results reveal that the educational interventions had an impact on the menstrual hygiene practices of the in-school adolescent girls in the three experimental groups, hence, the null hypothesis is rejected in favour of the alternative hypothesis (Table 4).

## Discussion

The present study shows that the age of participants across the four groups ranges from 10-19 years with the mean of 14.92 ± 1.82 years and majority of the girls being 14-16 which is comparable and similar to the age of high school adolescent girls reported in Saifai, Etawah, India [22], Saoner, Nagpur District, India [23] and Cross River State, Nigeria [24]. The mean age of menarche in the present study was 12.73 ± 1.38 years which is similar to the mean age reported among girls in Southwest Nigeria [25] but lower compared to findings conducted in District Sambhal, India [26]. The disparity in the mean age at menarche could be attributed to genetic, nutritional, and socioeconomic factors and general health; malnutrition, high level of physical activity, and chronic disease can as well delay menarche [22]. Hygienic-related practices are fundamental during menstruation as poor hygiene affects health by increasing vulnerability to infections of the reproductive and urinary tract [4-6]. The present study demonstrates that the level of menstrual hygiene practices of adolescent girls in the parent-led, peer-led, combination of parent and peer-led group, and the control group had a low level of practice of menstrual hygiene behaviors before the intervention. In the post-intervention, there was a significant improvement in the level of menstrual hygiene behaviours among the adolescent girls across the experimental groups compared to the control group. This finding agrees with the results of other studies in Bangladesh, Uyo, and Illorin, Nigeria [14,27,28]. There was also an increase in the mean scores of the adolescent girls´ menstrual hygiene practices at the 6^th^-week follow-up. This implies that students who underwent the educational intervention program had increased menstrual hygiene practices at the follow-up evaluation period. Therefore, it is appropriate to conclude that the educational intervention was effective in enhancing participants´ menstrual hygiene practices. Other researchers may develop interventions for out-of-school adolescents girls on menstrual hygiene practices and prevention of reproductive health infections.

**Limitations:**the findings of this study may be based on traditional, socio-economic, and cultural factors which influence the adoption of healthy behaviors in communities. The use of the findings of this study as a representation of adolescent girls in Nigeria may be limited due to the factors mentioned above.

## Conclusion

The study revealed significant differences in the level of adolescent girls´ menstrual hygiene practices between baseline and follow-up periods. The peer-led experimental group had the greatest change. This implies that the use of peers in the school setting was effective in impacting better practices of menstrual hygiene and this could be recruited for future intervention and be scaled up in other aspects of young girls´ reproductive health and well-being.

### What is known about this topic

- Hygiene-related practices are significant during menstruation to prevent being predisposed to reproductive tract infections and other complications;
- Majority of the global burden of reproductive tract infections lies in low- and middle-income countries especially in the rural settlements;
- Researchers have discovered between 31 and 56% of school girls in Nigeria using toilet tissue, cotton wool, or cloth to absorb menstrual blood as opposed to sanitary pads.

### What this study adds

- The study added that the level of practice of menstrual hygiene behaviors was lower at a baseline level of assessment and the post-intervention, had a significant improvement in the level of menstrual behaviours which at the follow-up, there were also significant changes;
- Having a theory-based and a combination of interventions is effective in influencing a change in behaviors as revealed by the findings of this study.
